# Ablation of atrial flutter related to the left atrial appendage occluder

**DOI:** 10.1016/j.hrcr.2025.02.011

**Published:** 2025-02-15

**Authors:** Chao Liu, Rongbing Peng, Manli Yu, Xinmiao Huang, Jiang Cao, Zhifu Guo

**Affiliations:** Department of Cardiovascular Medicine, Changhai Hospital, Naval Medical University, Shanghai, China

**Keywords:** Catheter ablation, Atrial fibrillation, Atrial flutter, Left atrial appendage occluder, Stroke

**Key Teaching Points**

• In patients with atrial flutter (AFL) who have an implanted occluder device, the atypical AFL is most likely attributable to atrial scar formation induced by the occluder disk.
• For the onset of AFL after left atrial appendage closure, mapping of the left atrial appendage region should be prioritized.
• Focal ablation should be the preferred strategy for AFL related to the left atrial appendage occluder. If focal ablation is unsatisfactory, linear ablation can be considered to achieve passive left atrial appendage electrical isolation.

## Introduction

Left atrial appendage closure (LAAC) has become the primary strategy to prevent stroke associated with atrial fibrillation (AF).^1^ Previous studies have indicated that the implantation of an occluder device can increase the risk of atrial arrhythmias in patients, potentially due to scarring caused by the device.^2^, ^3^, ^4^ We report a case of atrial flutter (AFL) related to LAA occluder, and tachycardia was terminated during focal ablation near the occluder disk.

### Case report

**Figure 1:**
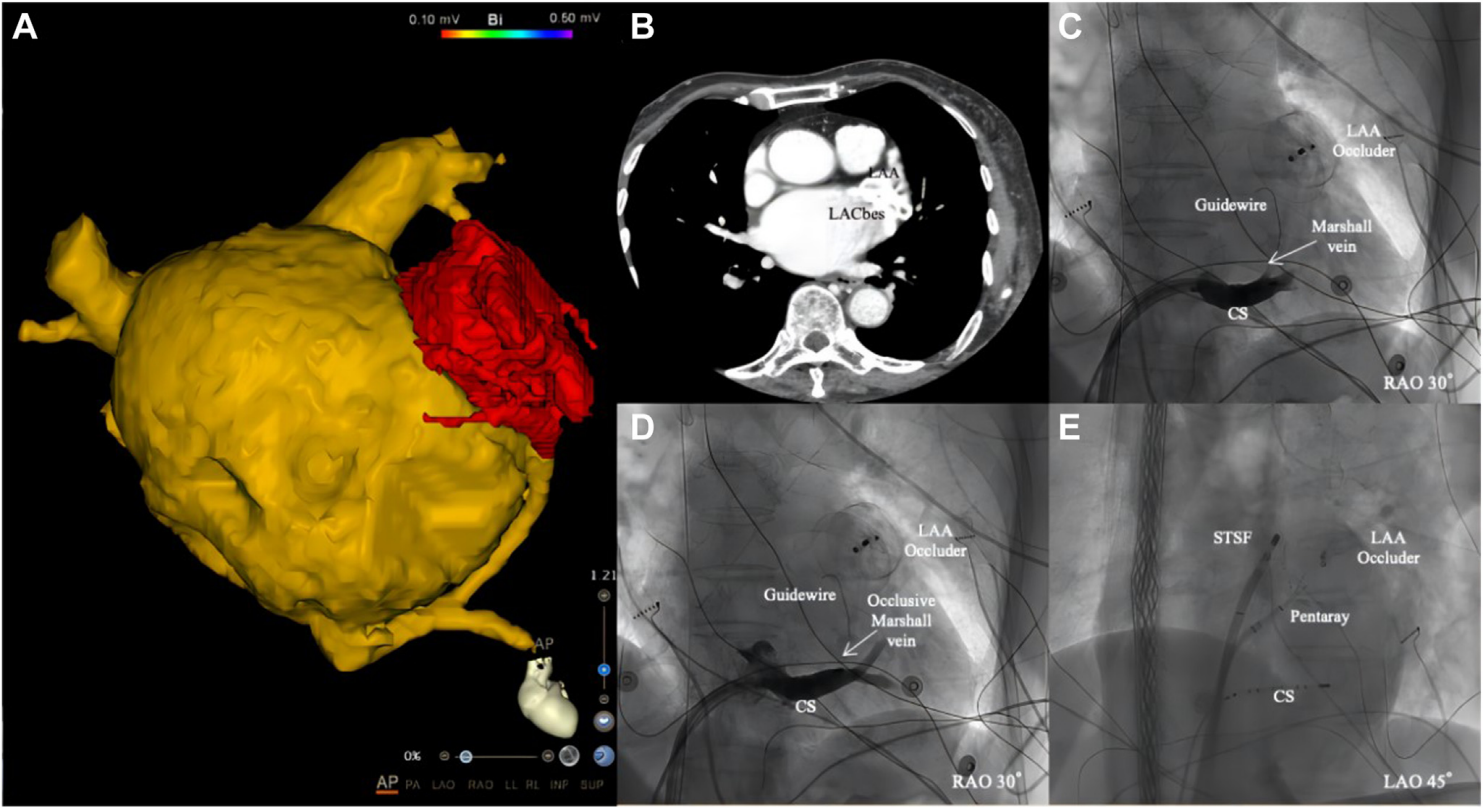
Left atrial appendage (LAA) occluder and images during ablation. **A:** 3-Dimensional model of left atrium with LAA occluder (*red area*). **B:** LAA occluder leakage. **C:** Marshall vein image. **D:** Marshall vein blocked after injection of anhydrous alcohol. **E:** Pentaray mapping catheter (Biosense Webster, Diamond Bar, CA) and STSF pressure contact ablation catheter (Biosense Webster). CS = coronary sinus.

An 82-year-old female patient presented with palpitations persisting for a week, and a 24-hour electrocardiogram revealed AF. The patient had previously undergone catheter ablation and left atrial appendage closure (LACbes 32 mm, Shanghai Pushmed; Figure 1A) in 2021 due to persistent AF and stroke. Consequently, she was advised to undergo a repeat catheter ablation.

Using the Seldinger technique, bilateral femoral veins were punctured, and a 10-pole catheter was placed in the coronary sinus. Due to the repeat ablation, the patient's age, and the LAA occluder leak (Figure 1B), we decided to use the “2C3L” ablation approach (ie, pulmonary vein isolation-roof-mitral and tricuspid isthmus line) to improve sinus rhythm maintenance. Marshall vein ethanol ablation was performed successfully (Figure 1C and 1D). After transseptal puncture, the mapping catheter (Pentaray, Biosense Webster, Diamond Bar, CA) and pressure contact ablation catheter (STSF, Biosense Webster) were delivered to the left atrium (LA) (Figure 1E).

**Figure 2:**
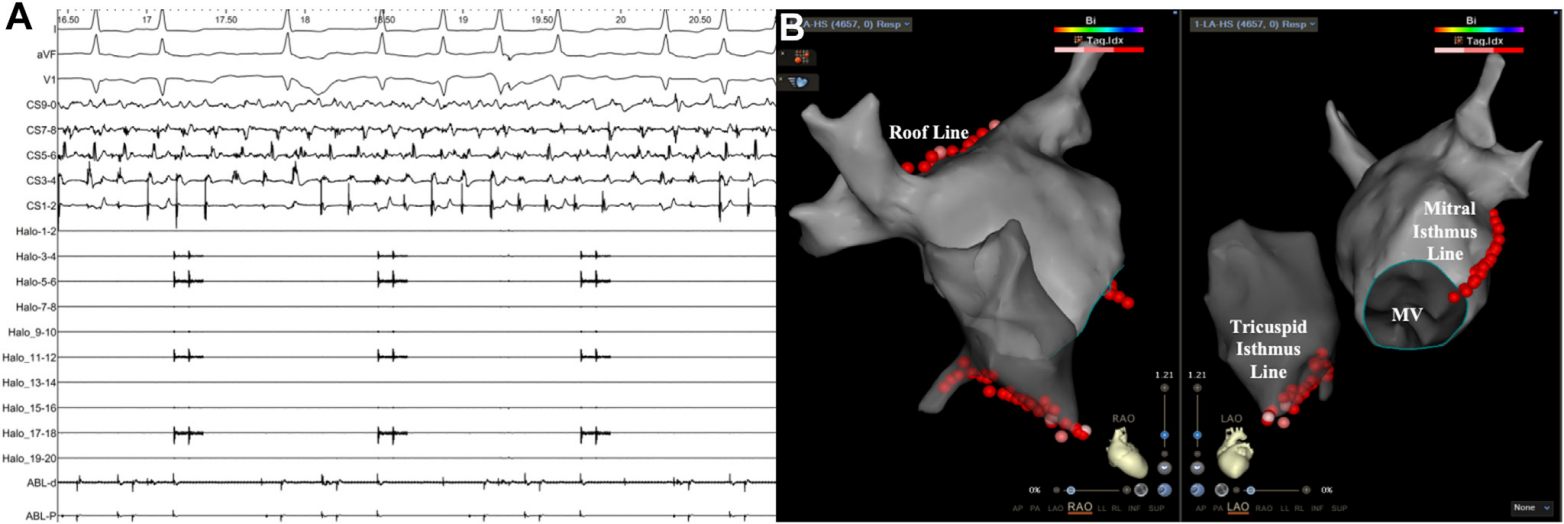
Intracardiac electrocardiogram (ECG) and 3-dimensional model of the left atrium. **A:** Initial intracardiac ECG indicated persistent atrial fibrillation. **B:** After electrical cardioversion converted to sinus rhythm, all pulmonary veins showed no potential reconnection. The “3L” ablation strategy was performed (ie, roof, mitral, and tricuspid isthmus line). MV = mitral valve.

**Figure 3:**
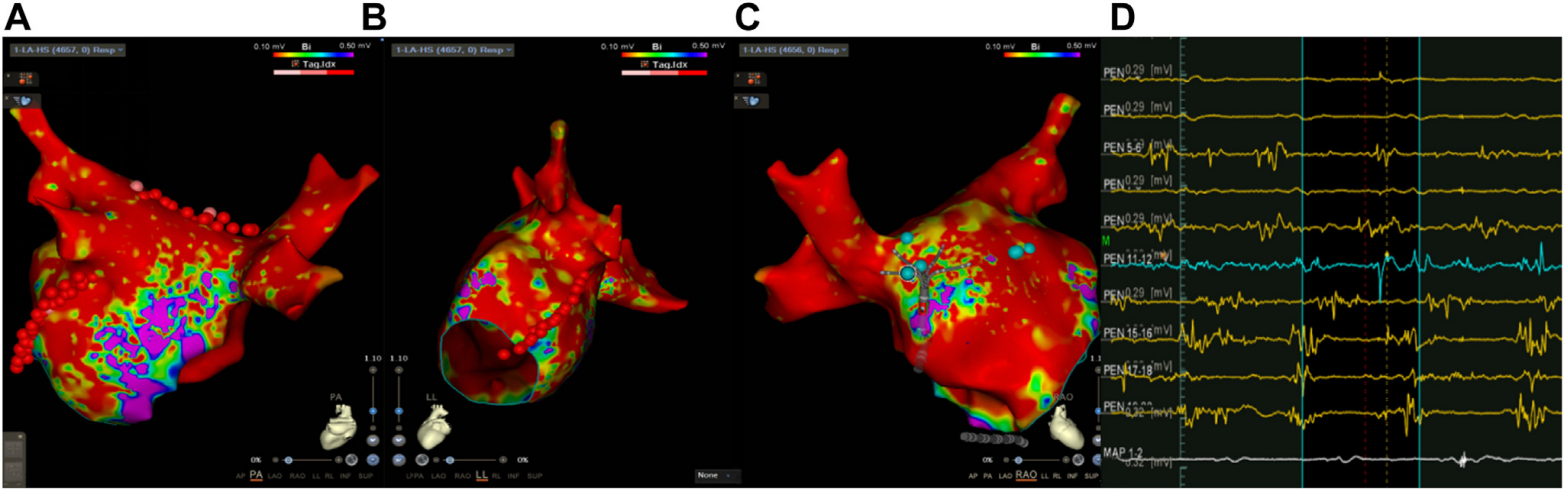
Voltage mapping model. **A–D:** Voltage mapping showed a large area of low voltage on the left atrium roof and anterior wall with complex fractionated atrial electrogram.

The intracardiac electrocardiogram initially showed AF (Figure 2A), which was later converted to sinus rhythm by electrical cardioversion. The LA model was constructed using the mapping catheter, revealing no potential recovery in all pulmonary veins. The STSF catheter (40–45 W, ablation index 400–450) was then used to perform the linear ablation of the roof, mitral isthmus, and tricuspid isthmus, verifying the blocked line (Figure 2B). Voltage mapping showed a large area of low voltage with complex fractionated atrial electrograms on the LA roof and anterior wall (Figure 3A–3D).

**Figure 4:**
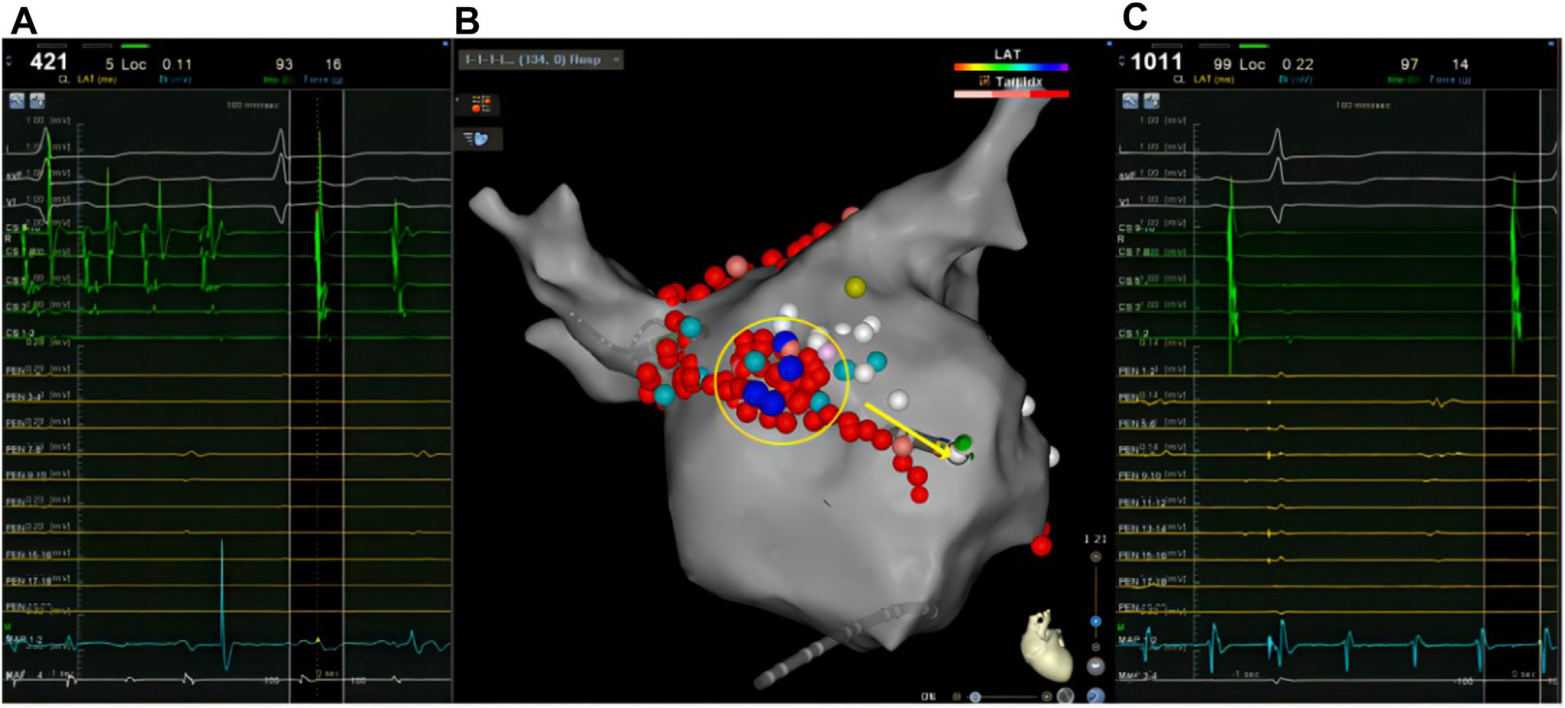
Ablation of the left atrium anterior wall. **A:** The atrial fibrillation terminated after focal ablation of the right pulmonary vein near the anterior wall and converted to atrial flutter (AFL) (*yellow circle*). **B:** Anterior wall linear ablation (*yellow arrow*). **C:** The AFL was terminated, the anterior wall line blocked, but tachycardia was still recorded on the ablation catheter.

**Figure 5:**
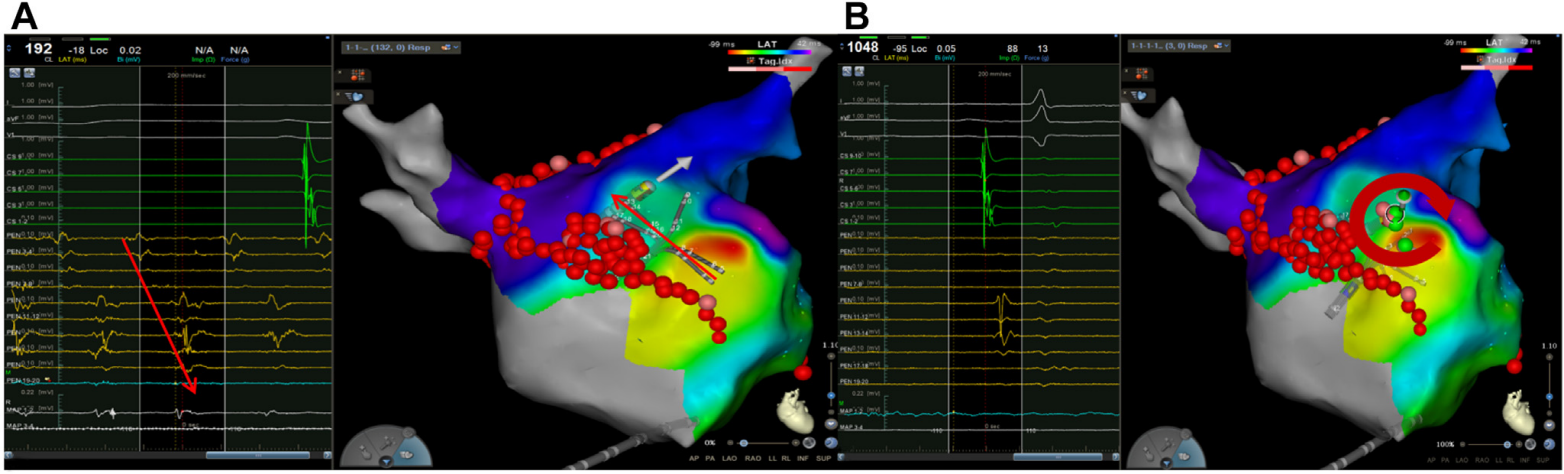
The ablation of atrial flutter (AFL) correlated left atrial appendage (LAA) occluder. **A:** The Pentaray mapping catheter (Biosense Webster, Diamond Bar, CA) recorded AFL around the LAA occluder, while the LA still maintained sinus rhythm. **B:** Activation mapping indicated the reentry of AFL around the LAA and occluder, which was terminated during local ablation, with autonomic potential observed near the LAA ostium.

Due to the spontaneous triggering of AF and the factors mentioned above, modified substrate ablation was performed on the anterior wall of the LA (Figure 4B). During the repeat ablation, AF stopped and converted into a new AFL (Figure 4A; cycle length = 421 ms). The anterior wall line was supplemented for ablation, terminating the AFL (Figure 4C) and blocking the ablation line. However, the catheter recorded AFL near the LAA, while the sinus rhythm was still maintained in the LA (Figure 4C). The Pentaray catheter was used to map the anterior wall of the LA and the LAA (Figure 5A), indicating that the reentrant circuits of AFL might be around the LAA and the occluder. The AFL was terminated during focal ablation around the LAA ostium, where an autonomic potential could be recorded (Figure 5B). After observing for 30 minutes, the intervention was completed once the linear block and the absence of tachycardia in the LAA were confirmed.

Due to the presence of leakage after LAAC, the patient still required long-term oral rivaroxaban 10 mg once per day and pantoprazole 40 mg once per day for 1 month to prevent injury to the esophagus. The patient was followed up for 1 year and maintained sinus rhythm without any stroke events.

## Discussion

We reported a case of recurrent AF after catheter ablation and LAAC 2 years prior. Despite using the 2C3L ablation strategy, AF was still triggered and later terminated using modified ablation at the LA anterior wall. Linear ablation of the anterior wall was undertaken due to the presence of AFL, successfully terminating the tachycardia when the anterior wall line was blocked. However, AFL was still mapped near the LAA occluder device. Although the LA maintained sinus rhythm, the reentrant circuits appeared to be around the LAA occluder, and tachycardia was stopped during focal ablation near the occluder.

LAAC has become the primary strategy to prevent stroke associated with AF.^1^ Previous studies have indicated that the implantation device can increase the risk of atrial arrhythmias, due to scarring caused by the device.^2^, ^3^, ^4^ This case was related to AFL associated with the occluder. The blocked lines in the roof, anterior, and posterior walls effectively isolated the LAA, leading to the blockage of AFL conduction in the LAA. The catheter subsequently recorded the reentrant circuits of AFL near the LAA region and successfully terminated them, further confirming the correlation between AFL and the LAA occluder. The AFL exhibited a leading coronary sinus 90, which could be associated with the blocked line of the mitral isthmus and roof in the LA. Lewalter and colleagues^4^ also reported a case of AFL associated with LAA occluder. They successfully terminated the tachycardia when mapping a region of slow conduction between the base of the LAA and the mitral valve, where ablation was performed. This atypical AFL was most likely related to implant-related atrial scarring and similar with our case. Therefore, for patients with AFL implanted with an occluder device, including but not limited to LAA, activation mapping of the occluder device area was essential. Nonetheless, there are several limitations to this case. Firstly, mapping of the AFL should have been prioritized rather than performing ablation of the anterior wall line. If the reentrant circuit had been mapped near the LAA, only local ablation would have been necessary. The ablation of the anterior wall line may have caused delayed conduction in the LAA, combined with passive LAA electrical isolation (LAAEI).^5^ Previous studies suggest that LAAEI could increase the risk of stroke.^5^^,^^6^ Nevertheless, given the patient's implantation of the LAA occluder and long-term anticoagulation therapy, the risk of stroke was relatively low. This case offers electrophysiologists a new perspective on AFL after LAAC, emphasizing the need to prioritize mapping the area around the LAA occluder. Focal ablation should be the preferred strategy whenever possible. If focal ablation is unsatisfactory, linear ablation can be considered to achieve passive LAAEI.

## Conclusion

In patients with AFL who have an implanted occluder device, the atypical AFL is most likely attributable to atrial scar formation induced by the occluder disk, and mapping of the LAA region should be prioritized. If focal ablation is unsatisfactory, linear ablation can be considered to achieve passive LAAEI.

## Disclosures

The authors have no conflicts of interest to disclose.

## References

[1] Ulf L., Carsten S., Apostolos T. (2024). Left atrial appendage closure for stroke prevention in atrial fibrillation: current status and perspectives. Eur Heart J.

[2] Steckman D.A., Nguyen D.T., Sauer W.H. (2014). Catheter ablation of atrial fibrillation and left atrial flutter in a patient with a left atrial appendage occlusion device. Europace.

[3] Reinder E., Manon R., Charlotte H. (2020). Atrial fibrillation in patients with an atrial septal defect in a single centre cohort during a long clinical follow-up: its association with closure and outcome of therapy. Open Heart.

[4] Thorsten L., Klaus T., Tobias G. (2021). Successful catheter ablation of a left atrial appendage occluder-related atypical atrial flutter. J Interv Card Electrophysiol.

[5] Andreas R., Roland R.T., Tina L. (2016). Unexpectedly high incidence of stroke and left atrial appendage thrombus formation after electrical isolation of the left atrial appendage for the treatment of atrial tachyarrhythmias. Circ Arrhythm Electrophysiol.

[6] Luigi D.B., Sanghamitra M., Chintan T. (2019). Stroke risk in patients with atrial fibrillation undergoing electrical isolation of the left atrial appendage. J Am Coll Cardiol.

